# NMDA receptor mediated tonic excitatory currents are elicited by EAAT inhibition on human neocortical pyramidal neurons

**DOI:** 10.3389/fnsyn.2026.1844197

**Published:** 2026-06-05

**Authors:** Béla Márton, Levente Ecsedi, Krisztina Pocsai, Abdennour Douida, Karri Lamsa, Gábor Hutóczki, Álmos Klekner, Balázs Pál

**Affiliations:** 1 Department of Physiology, Faculty of Medicine, University of Debrecen, Debrecen, Hungary; 2 Doctoral School of Molecular Medicine, University of Debrecen, Debrecen, Hungary; 3 Hungarian Centre of Excellence for Molecular Medicine (HCEMM), Szeged, Hungary; 4 Neurosurgery Clinic, Clinical Center, University of Debrecen, Debrecen, Hungary

**Keywords:** excitatory postsynaptic current, neocortex, NMDA receptor, pyramidal neuron, tonic inward current

## Abstract

**Introduction:**

Ambient glutamate is capable of regulating neural excitability and contributes to several brain pathologies. It is well established by experiments on rodent tissue that extrasynaptic glutamate –mainly regulated by astrocytic glutamate uptake- can elicit both phasic slow inward currents (SICs) and tonic inward currents. These currents are thought to be mediated by overlapping mechanisms and receptors; and exist under physiological conditions. SICs were also found in the human neocortex but the existence of the tonic inward currents and their relationship with SICs have not been demonstrated yet.

**Methods:**

We aimed to investigate tonic inward currents and their relation to slow inward currents (SICs) elicited by the inhibition of glutamate transport in the human neocortex. Brain samples removed for accessing the primary brain tumors or metastases were collected from patients with broad age range. Slice electrophysiological approach and post-hoc morphological analysis was used.

**Results:**

We found that inhibition of the EAAT transporters by DL-TBOA elicited both SICs and tonic inward currents. The tonic current was only partially reverted by the GluN2B subunit specific NMDA receptor (NMDAR) antagonist ifenprodil, whereas SICs were almost fully eliminated. The amplitude of the current was inversely proportional with the age of the patient and disappeared in elderly over the age of 70. The charge transfer of SICs per minute was directly proportional with the magnitude of the tonic inward current. Omitting magnesium from the recording solution or application of the EAAT positive allosteric modulator GT949 did not elicit any notable tonic current.

**Discussion:**

In summary, NMDAR-dependent tonic inward currents are overlapping but partially separable phenomena from SICs. One might hypothesize that the tonic current is only present under excitotoxic conditions and do not determine physiological neuronal excitability in human.

## Introduction

Glutamate is the most abundant neurotransmitter of the central nervous system (CNS) as cca. 70% of the synapses is glutamatergic. Its ionotropic receptors are the *N*-methyl-D-aspartate receptors (NMDARs) and non-NMDA receptors, from which the subgroup with the greatest importance is the 2-amino-3-(3-hydroxy-5-methyl-isoxazol-4-yl)propanoic acid receptor (AMPAR; Paoletti et al., 2013; Nusser, 2000). Metabotropic glutamate receptors (mGluRs) also have significant and diverse roles in modulation of CNS functions (Nicoletti et al., 2011).

The presence, actions and receptors of glutamate are not restricted to the synaptic cleft but to the extrasynaptic space as well. Extrasynaptic glutamate has magnitudes lower concentration (in contrast to the millimolar concentration of the synaptic cleft, it is in the micromolar-nanomolar range; Kessler, 2013; van der Zeyden et al., 2008), but it is capable of activating extrasynaptic NMDARs, AMPARs and mGluRs (Harris and Pettit, 2007; Kopach and Voitenko, 2013; Nicoletti et al., 2011), which might differ from the synaptic ones in subunit composition (as NMDARs often possess the GluN2B subunit, Paoletti et al., 2013; Stroebel et al., 2018). The extrasynaptic glutamate has various physiological functions such as setting neuronal excitability and synaptic strength (see Pál, 2018). Its involvement in aging and various brain pathologies (as, e.g., stroke, epilepsy, malignancies or Alzheimer disease) is also widely described (see Pál, 2018).

Astrocytes have a pivotal role in regulating extrasynaptic glutamate. A recently identified astrocytic subgroup can release glutamate with vesicular exocytosis (De Ceglia et al., 2023), but various other mechanisms of glutamate release (as connexon/pannexon hemichannels, ionotropic purinergic receptors, organic anion transporters, cystine-glutamate antiport, volume-regulated anion channels and bestrophin-1 channel) also exist (see Pál, 2018). Besides releasing glutamate, astrocytes have an outstanding role in its clearance. Although neurons also possess glutamate transporters (the excitatory amino acid transporters (EAATs) 3 and-4; Holmseth et al., 2012; Dehnes et al., 1998), the astrocytic uptake via EAAT1 (glutamate–aspartate transporter, GLAST) and EAAT2 (glutamate transporter 1; GLT1) is responsible for the vast majority of the glutamate clearance (Vandenberg and Ryan, 2013).

We previously found that human neocortical pyramidal neurons are targets of extrasynaptic glutamatergic actions. Activation of GluN2B-containing NMDARs elicited SICs on them. These currents contributed to synaptic plasticity and declined with age. This age-dependent decline was in accordance with the decrease in the GluN2B subunit immunolabelling (Csemer et al., 2023). It is also well described that –besides the phasic SICs- neuronal tonic inward currents might be elicited by the extrasynaptic glutamate on the rodent tissue (Angulo et al., 2004). These currents were thought to be related to SICs and existed under control conditions as well. However, some questions remained unaddressed. First, whether the tonic inward currents exist on human pyramidal cells and not only in rodents. Second, whether mechanisms mediating them and the SICs are overlapping or distinct. Third, whether tonic inward currents are present under control conditions without manipulations of the glutamate uptake. Fourth, the impact of tonic inward currents on synaptic currents was investigated.

We recorded the tonic inward currents provoked by the blockade of the glutamate uptake on human pyramidal neurons and found that this phenomenon does not exist without inhibition of glutamate uptake. In contrast to SICs, tonic inward currents are not (only) mediated by GluN2B subunit containing NMDA receptors but by ones with different subunit composition. The appearance of these currents correlated well with SIC activity and had an age-dependent decline. Tonic inward currents were responsible for the increase in the excitability of the whole neuronal network but likely did not directly affect synaptic strength.

## Materials and methods

### Solutions, chemicals

For *ex vivo* recordings, artificial cerebrospinal fluid (aCSF) was employed with the following composition (in mM): NaCl, 120; KCl, 2.5; NaHCO_3_, 26; glucose, 10; NaH_2_PO_4_, 1.25; myo-inositol, 3; ascorbic acid, 0.5; sodium-pyruvate, 2; CaCl_2_, 2; MgCl_2_, 1; pH 7.4. When SICs and tonic currents were recorded, nominally magnesium-free medium was made by omitting MgCl_2_. For slice preparation, 95 mM NaCl was replaced by glycerol (60 mM) and sucrose (130 mM). All chemicals listed above were purchased from Sigma-Aldrich (St. Louis, MO, USA).

### Human samples

Involvement of human brain samples in experiments was performed in accordance with national and international guidelines. Protocols were approved by the Hungarian National Public Health and Medical Officer Center and the regional and national Medical Research Councils (DE KK RKEB/IKEB 4259–2014, RKEB 6633–2023, HD-01-GBM, HBR/052/00130–1/2015).

Samples from 25 patients were included. The tissue from 8 patients (38–74 years old, both genders) were involved in experiments with DL-TBOA, 7 patients (40–79, both genders) in GT949 experiments. With a single exception (AV malformation), all patients were operated with primary brain tumors or metastases. Intact neocortical tissues, removed during the approach of the tumor or for prevention of herniation, were collected. Samples where any signs of tumor infiltration were seen were excluded from analysis (if the tumor cells or lymphocytes visibly appeared or in case of reduction of neuronal action potential amplitude or firing rate; Table 1). The samples were collected in the operating theatre and transferred to the Department of Physiology in oxygenated ice-cold low Na^+^ aCSF within 10 min after removal. In the laboratory, the tissue was sliced to 350 μm thick pieces perpendicular to the brain surface (see below).

**Table 1:** Patient data.

**Age (yrs)**	**Gender**	**Location**	**Diagnosis**
Experiments with DL-TBOA			
38	♀	Right parietal	Glioblastoma multiforme
45	♀	Right temporal cortex	Glioblastoma multiforme
59	♀	Right occipital	Carcinoma metastasis (breast cancer)
59	♂	Parietal	Glioblastoma multiforme
61	♀	Right frontal	Carcinoma metastasis (small cell neuroendocrine lung cancer)
63	♀	Right frontal	Carcinoma metastasis (lung cancer)
70	♂	Right frontal	Carcinoma metastasis (stomach cancer)
74	♂	Right frontal	Carcinoma metastasis (lung cancer)
Experiments with GT949			
40	♀	Left occipital	Carcinoma metastasis (endometrium)
44	♀	Right frontal	AV malformation
55	♀	Right temporal	Glioblastoma multiforme
72	♂	Left frontal	Glioblastoma multiforme
77	♀	Right frontal	Carcinoma metastasis (lung cancer)
77	♂	Right frontal	Carcinoma metastasis (unknown origin)
79	♀	Left parietal	Carcinoma metastasis (unknown origin)

### Electrophysiology

Randomly chosen neocortical pyramidal neurons from layer II-III were recorded. The resistance of the patch pipettes was 6–8 MΩ, filled with pipette solution contained (in mM): K-gluconate, 120; NaCl, 5; 4-(2-hydroxyethyl)-1- piperazineethanesulfonic acid (HEPES), 10; Na_2_- phosphocreatinine, 10; ethylene glycol-bis(*β*-aminoethyl ether)-N, N, N′, N′-tetraacetic acid (EGTA), 2; CaCl_2_, 0.1; Mg-ATP, 5; Na_3_-GTP, 0.3; biocytin, 8; pH 7.3. Whole cell patch clamp experiments were performed at room temperature (23–25 °C) with an Axopatch 200A amplifier (Molecular Devices, Union City, CA, USA). Data were recorded with Clampex 10.0 software (Molecular Devices, Union City, CA, USA). Data analysis was performed with Clampfit 10.0 (Molecular Devices) and Synaptosoft MiniAnalysis (Synaptosoft, Decatur, GA, USA) software. Recordings with 20 MΩ series resistance or higher, with more than 10% change were excluded and only stable recordings with minimal leak currents and with under 10 pA spontaneous holding current fluctuation were considered.

Gap-free recordings for detecting SICs, holding currents and sEPSCs were performed under voltage-clamp configuration, at −60 mV holding potential. Recordings were started in naCSF and then it was replaced with nominally magnesium-free aCSF. For inhibition of (mainly astrocytic) glutamate uptake, 100 μM DL-*threo*-*β*-benzyloxyaspartic acid (DL-TBOA, Tocris Cookson Ltd., Bristol, UK, Jabaudon et al., 1999; Shimamoto et al., 1998), 5 μM ifenprodil (GluN2B-specific inhibitor, Tocris Cookson Ltd., Bristol, UK, Nuno-Perez et al., 2021; Reynolds and Miller, 1989) and 10 μM D-AP5 (nonspecific inhibitor, Tocris Cookson Ltd., Bristol, UK, Davies and Watkins, 1982; Csemer et al., 2023) were administered. The 3-((4-cyclohexylpiperazin-1-yl)(1-phenethyl-1*H*-tetrazol-5-yl)methyl)-6-methoxyquinolin-2(1*H*)-one (GT949, positive allosteric modulator of EAAT transporters, 10 μM, Kortagere et al., 2018) was also applied in another set of experiments.

At the end of all recordings of SICs or EPSCs, a cocktail of NBQX (10 μM), D-AP5 (10 μM), strychnine (1 μM), bicuculline (10 μM) was used to inhibit SICs and fast synaptic neurotransmission (Csemer et al., 2023).

SICs were identified as shown in our previous paper (Csemer et al., 2023): events with rise time longer than 20 ms were considered as SICs. The holding currents were measured as the peak of the frequency histogram from a 1-min-long recording (Kovács et al., 2017). Tonic currents elicited by the pharmacological agents were assessed in comparison with the spontaneous fluctuation of the holding current (the „baseline”) at the previous experimental arrangement free of pharmacons (in naCSF or in magnesium-free aCSF). This fluctuation was calculated as the difference of the frequency histogram peaks prior to drug application and 5 min before it.

### Morphological evaluation of recorded neurons

Neurons from electrophysiological experiments were labelled with biocytin of the pipette solution. After fixation (4% paraformaldehyde in 0.1 M phosphate buffer; *pH* 7.4; 4 °C), permeabilization was achieved with Tris buffered saline (in mM, Tris base, 8; Trisma HCl, 42; NaCl, 150; *pH* 7.4) containing 0.1% Triton X-100 and 10% bovine serum for 60 min. Next, phosphate buffer supplemented with streptavidin-conjugated Alexa488 (1:300; Molecular Probes Inc., Eugene, OR, USA) was applied for 90 min.

The Alexa488 signal was visualized under confocal microscope (Zeiss LSM 510; Carl Zeiss AG); tile scan images were taken with 40x objective and 1 μm z stacks.

All data represented as mean ± SEM. Normality tests were used to determine the normal distribution of datasets. Statistical comparison of two datasets was assessed with two sample Student’s t test, whereas multiple comparisons were performed using Tukey’s multiple comparisons test. Changes were considered significant when *p* < 0.05. Pearson’s correlation coefficient (*r*) and the *p*-value from the linear regression test were applied to assess correlations between parameters by plotting them against each other followed by the linear fit of the dataset.

## Results

**Figure 1:**
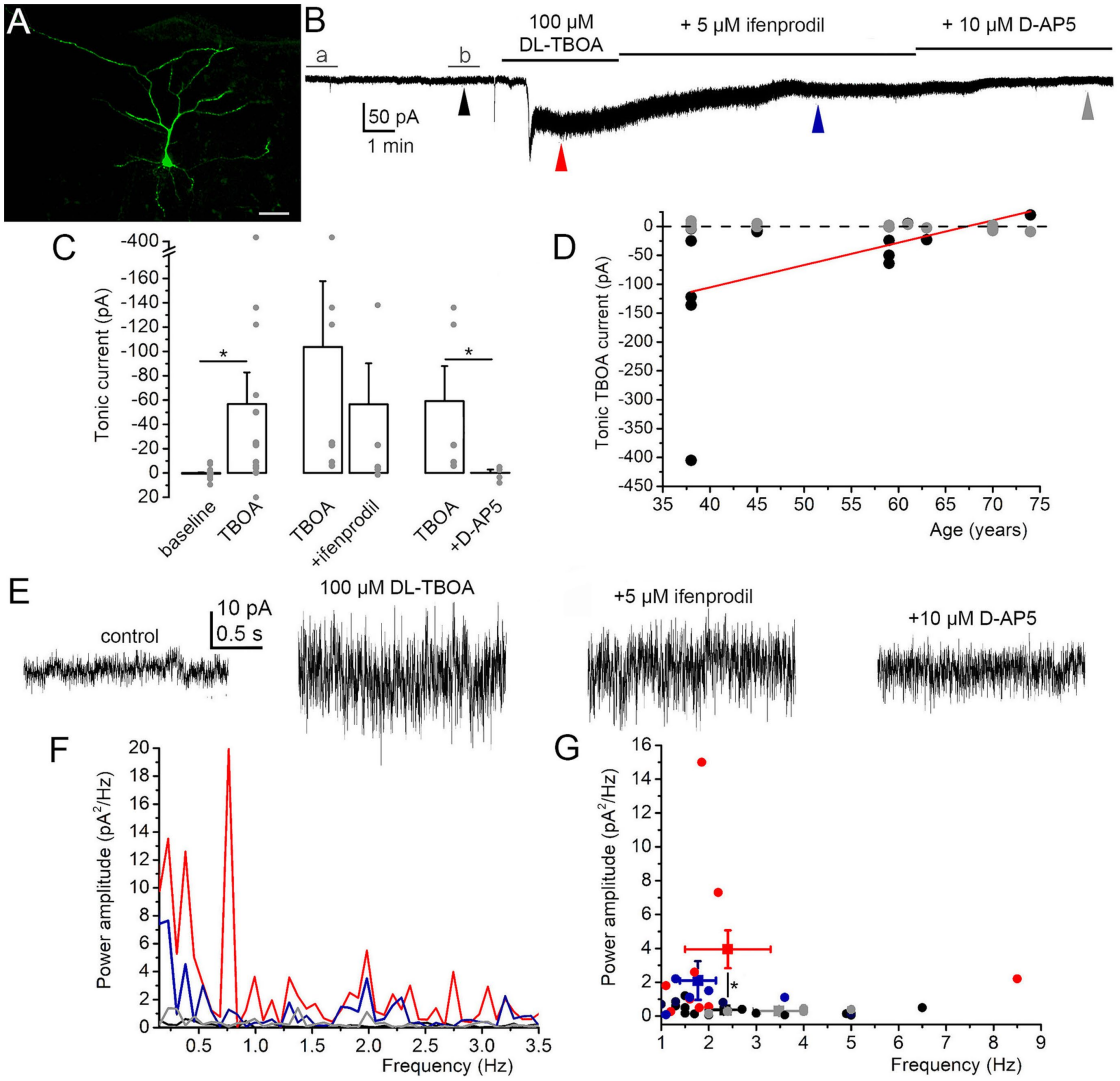
Inhibition of glutamate uptake elicits N-methyl-D-aspartate receptor- (NMDAR-) mediated tonic inward current. **(A)** A pyramidal neuron labelled with biocytin. Scale bar: 50 μm. **(B)** A representative current trace of an experiment using DL-TBOA (100 μM) and additional ifenprodil (5 μM) and D-AP5 (10 μM). **(C)** Statistical analysis on the glutamate transporter inhibition and its reversion by the GluN2B subunit specific blocker ifenprodil and the non-specific NMDAR antagonist D-AP5. Columns and error bars: average ± SEM, gray dots: individual data. **(D)** Age-dependence of the tonic inward current elicited by DL-TBOA. Black dots: individual data, gray dots: baseline fluctuation calculated from the condition prior to drug application [indicated with gray lines and letters (a, b) on **(A)**], red line: linear fit of the black dots, dashed line: no change (*p* = 0.037; *r*^2^ = 0.26). (**E**) Oscillation of the baseline under control conditions, with DL-TBOA and with additional ifenprodil or D-AP5. The corresponding arrows on **(A)** indicate the locations of the traces on **(E)** (black: control, red: DL-TBOA, blue: ifenprodil, gray: D-AP5). **(F)** Power spectra of the representative baseline oscillations under control conditions (black), with DL-TBOA (red), with additional ifenprodil (blue) and D-AP5 (gray). **(G)** Statistical analysis of the power amplitudes plotted against the frequency belonging to them. Squares and error bars: averages ± SEM, dots: individual data. Black: control, red: DL-TBOA, blue: ifenprodil, gray: D-AP5. *p < 0.05, **p < 0.01, ***p < 0.001.

In the first set of experiments, human neocortical pyramidal neurons were patched and gap-free recordings were performed in voltage-clamp configuration, with a holding potential of −60 mV. Pyramidal neurons of layers II-III were labelled with biocytin and photomicrographs were taken after *post hoc* recovery (Figure 1A). After recording in naCSF and magnesium-free aCSF, the magnesium-free solution was supplemented with the EAAT inhibitor DL-TBOA (100 μM; n = 16), the GluN2B subunit specific NMDA receptor antagonist ifenprodil (5 μM) and the non-specific NMDA receptor antagonist D-AP5 (10 μM; Figure 1B) concomitantly. Only those recordings were considered, where the spontaneous fluctuation of the holding current did not exceed 10 pA. In 10 cases, a tonic inward current greater than −5 pA was measured, whereas an outward current of +20 pA was detected in a single case. In 5 further cases, the measured tonic current did not exceed the chosen cutoff value (5 pA). The tonic current elicited by DL-TBOA (−56.8 ± 25.88 pA) was significantly greater than the spontaneous fluctuation of the holding current in magnesium-free aCSF (0.55 ± 1.13 pA; *n* = 16; *p* = 0.02; Figures 1B–E). In 7 cases, additional ifenprodil was applied. In 4 of these cases, the tonic inward currents by TBOA was successfully reverted. However, because of the further 3 cases with no reversion, the differences in the tonic currents were statistically non-significant (−103.7 ± 54.2 pA in TBOA, −56.64 ± 33.57 pA in ifenprodil, *p* = 0.24; Figure 1C). In 5 cases, the additionally administered D-AP5 fully reverted the inward current (−59.2 ± 28.72 pA in TBOA, −0.3 ± 2.59 pA in D-AP5, *p* = 0.037; Figure 1C).

The magnitude of tonic currents elicited by TBOA correlated well with the age: greater currents were recorded samples of younger patients, and a decline with age could be observed (Figure 1D; *p* = 0.037; *r*^2^ = 0.26). Above the age of 70, tonic inward currents were not detected.

We also observed that application of DL-TBOA elicited a baseline noise which was successfully inhibited by the NMDAR antagonist D-AP5 (Figures 1E,F). Analyzing the power spectra of the baseline oscillations, we found that the power amplitude was significantly increased with DL-TBOA (from 0.37 ± 0.1 of control to 3.95 ± 1.13 pA^2^/Hz with TBOA, *p* = 0.0148; 2.1 ± 1.14 pA^2^/Hz with additional ifenprodil and 0.31 ± 0.06 pA^2^/Hz with D-AP5), whereas no significant change of the frequency belonging to the power maximum was seen (2.34 ± 0.42 pA^2^/Hz in control, 2.4 ± 0.91 pA^2^/Hz in TBOA, 1.77 ± 0.38 pA^2^/Hz in ifenprodil, 3.48 ± 0.56 pA^2^/Hz in D-AP5; Figure 1G).

**Figure 2:**
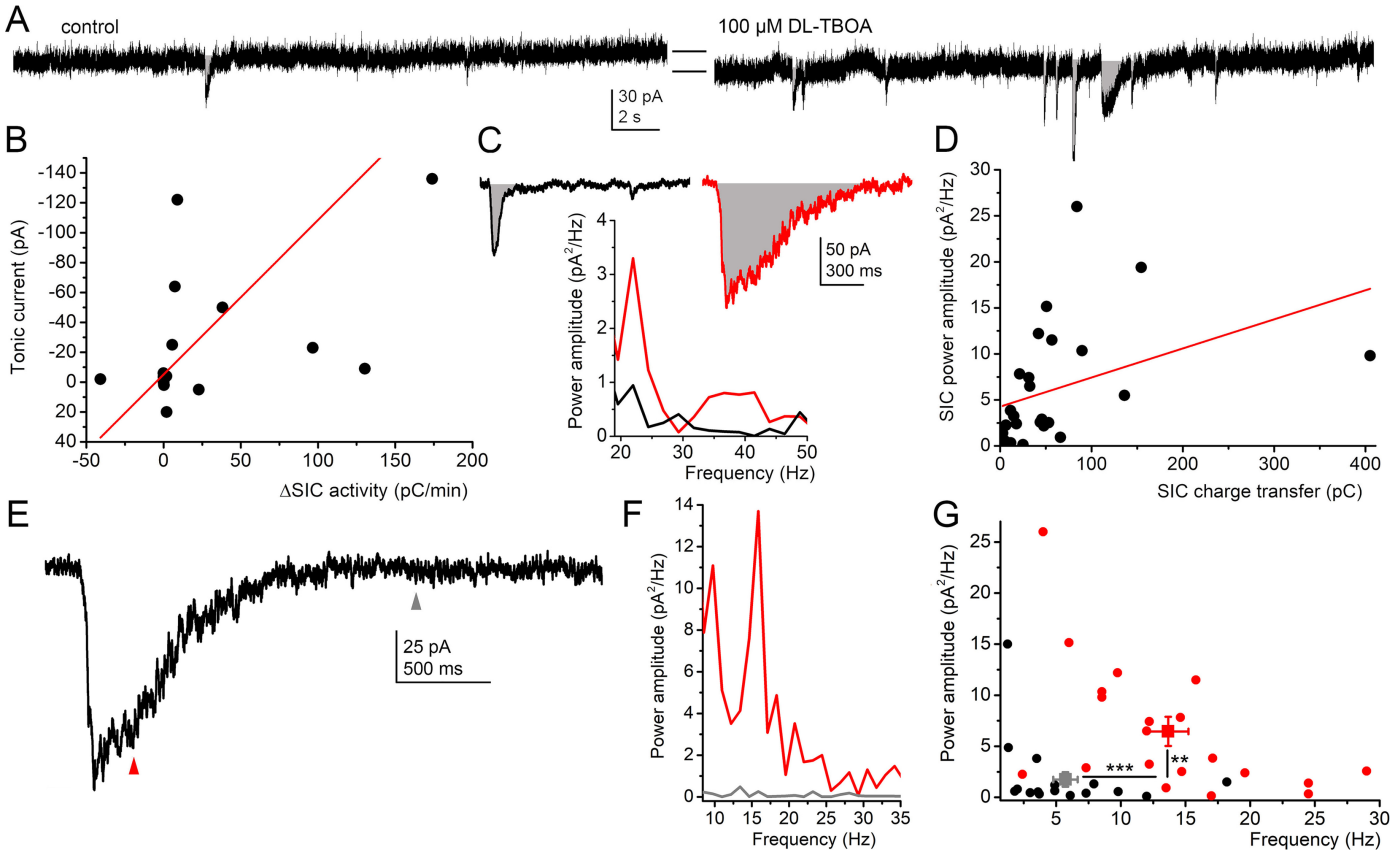
The amplitude of the tonic inward current correlates with changes in charge transfer by slow inward currents (SICs). **(A)** Representative current traces under control conditions and with DL-TBOA. The lines between the traces indicate the magnitude of the shift in the baseline, the gray areas represent the charge transfer by SICs. **(B)** The tonic current elicited by DL-TBOA correlates well with the changes in SIC activity (charge transfer per minute). Black dots: individual data, red line: linear fit (*p* < 0.0001, *r*^2^ = 0.636)**. (C)** The oscillatory activity of the declining phase of SICs correlate well with the charge transfer by SICs. Black trace: representative SIC recording with low charge transfer, red trace: SIC recording with great charge transfer, bottom panel: power spectra of the declining phases shown above with the same color code. **(D)** Correlation analysis between the charge transfer by SICs and the power amplitudes of the declining phases. Black dots: individual data, red line: linear fit (*p* = 0.0479, *r*^2^ = 0.153). **(E)** A representative current trace with two concomitant SICs under DL-TBOA application. Red, gray arrows: locations of power spectrum measurements of **(F)**. **(F)** Power spectra of the representative trace on **(E)** (red: declining phase of the SIC, gray: holding current). **(G)** Differences of the maximal power amplitudes and frequencies belonging to it between SICs and tonic currents. Red square and error bars: average of SIC declining phase, gray square and error bars: average of tonic currents, red dots: individual data of SICs, black dots: individual data of tonic currents (*p* < 0.0001 for the frequency, *p* = 0.00234 for the power amplitude). *p < 0.05, **p < 0.01, ***p < 0.001.

The charge transfer by SICs per minute (named as ‘SIC activity’) was also increased by application of DL-TBOA (Csemer et al., 2023). By re-analyzing the SIC data from our previous paper, we correlated the tonic inward currents with changes in SIC activity by TBOA and found a strong correlation between them: increased SIC activity was directly proportional with the tonic currents (*p* < 0.0001; *r*^2^ = 0.636; *n* = 19; Figures 2A,B). Regarding oscillatory activity of the declining phases of SICs and the charge transfer of SICs, a weaker but existing correlation was seen: the greater the SIC charge transfer was, the greater oscillatory power amplitude was found (*p* = 0.0479; *r*^2^ = 0.153; *n* = 19; Figures 2C,D).

The oscillatory activity of the declining phases of SICs and the tonic currents elicited by DL-TBOA had different power amplitude and frequency. The tonic current had 1.75 ± 0.71 pA^2^/Hz power amplitude and 5.72 ± 0.94 Hz frequency belonging to it, whereas the declining phase of SICs had a significantly greater oscillatory activity with a power amplitude of 6.46 ± 1.43 pA^2^/Hz power amplitude and 13.66 ± 1.56 Hz frequency (*p* = 0.00234 for the power amplitude and *p* < 0.0001 for the frequency; *n* = 20; Figures 2E–G).

**Figure 3:**
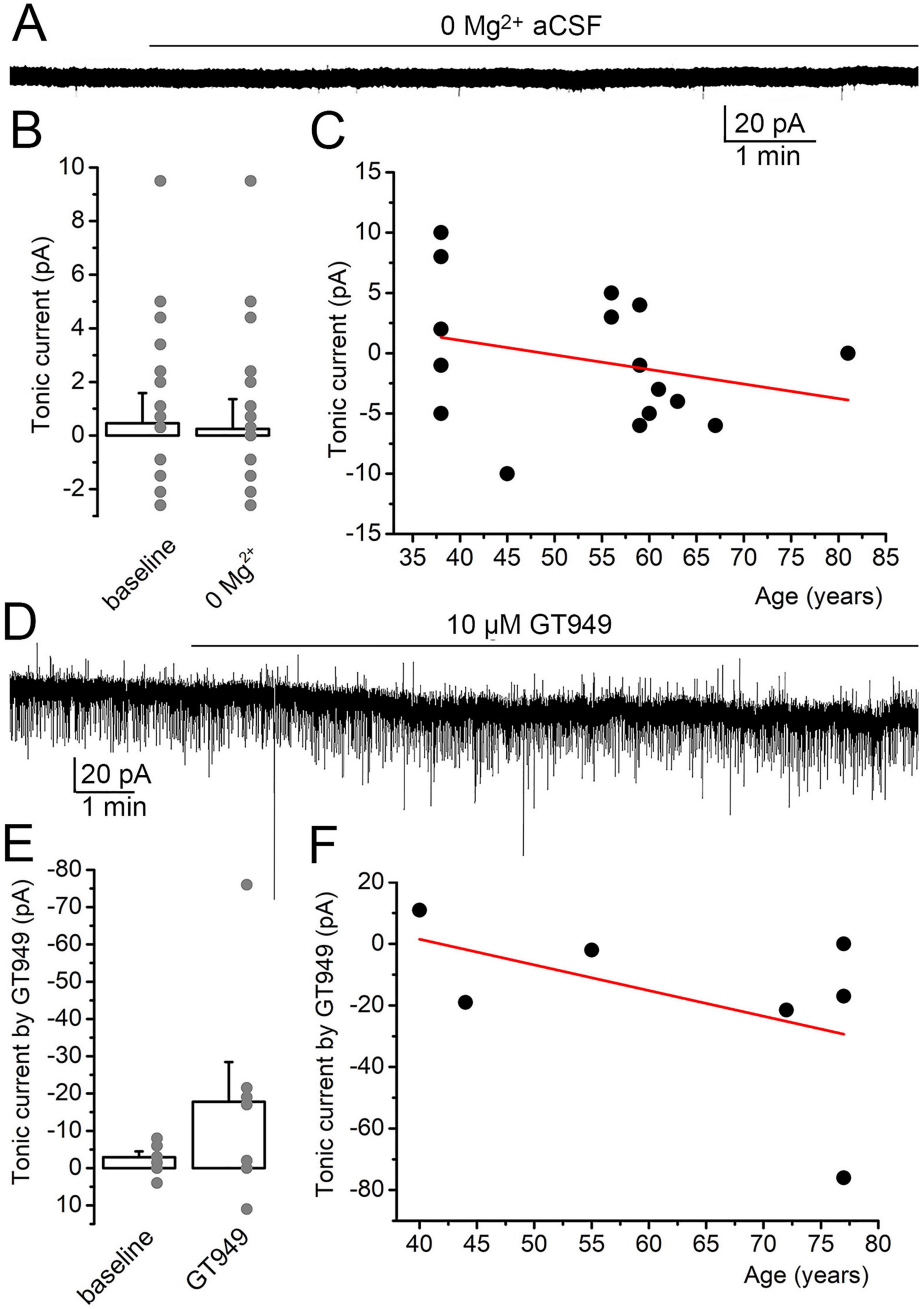
No tonic current is elicited by shifting to magnesium-free conditions or facilitating glutamate uptake. **(A)** Representative current trace with washing in and application of nominally magnesium-free artificial cerebrospinal fluid (aCSF). **(B)** Statistical comparison of the baseline fluctuation in normal aCSF (control conditions) and the current shift with application of magnesium-free aCSF. Columns: average ± SEM, gray dots: individual data points. No significant difference was seen. **(C)** No age-dependence can be revealed in baseline shifts elicited by magnesium-free solutions. Black dots: individual data, red line: linear fit (*p* = 0.29, *r*^2^ = 0.079). **(D)** Representative current trace with application of 10 μM GT949 (positive allosteric modulator of excitatory amino acid transporters; EAATs). **(E)** Statistical comparison of the baseline fluctuation in normal aCSF (control conditions) and the current shift with application of GT949. Columns: average ± SEM, gray dots: individual data points. No significant difference was found. **(F)** No age-dependence of the tonic currents elicited by GT949 was seen. Black dots: individual data, red line: linear fit (*p* = 0.23, *r*^2^ = 0.269). *p < 0.05, **p < 0.01, ***p < 0.001.

We previously showed that omitting magnesium from the recording solution significantly and drastically increased SIC activity by removing magnesium blockade of the NMDARs (Csemer et al., 2023). In the present project, we re-analyzed the previous data to assess whether magnesium-free conditions can elicit similar tonic inward currents as seen with DL-TBOA. No inward current was seen by the removal of magnesium blockade (0.46 ± 1.13 pA in naCSF and 0.25 ± 1.11 pA in magnesium-free aCSF; *n* = 16; Figures 3A,B). No age-dependence of the tonic currents elicited by the magnesium-free solution was revealed (*p* = 0.29; *r*^2^ = 0.079; Figure 3C).

When the positive allosteric modulator GT949 was applied, appearance of a tonic outward current was expected by the inhibition of a tonic inward current present under control conditions. On the contrary, similar to the magnesium-free conditions, no tonic current significantly different from the baseline fluctuation was found (−2.93 ± 1.57 pA in zero magnesium control and −17.78 ± 10.69 with additional TBOA; *p* = 0.097; *n* = 7; Figures 3D,E). Similar to above, no age-dependence of GT949 actions on the tonic currents was revealed (*p* = 0.269; *r*^2^ = 0.23; Figure 3F).

We also expected that GT949 fully inhibits SIC activity as these events are consequences of the non-synaptic glutamate release; and supposed to disappear if the ambient glutamate level is decreased by enhanced glutamate uptake. In 4 cases from 7, this inhibition of SICs was well seen but extreme SIC activity was provoked by GT949 in a single case. The patient history belonging to this singe case was not different from the others: the tissue was free of glioblastoma infiltration and no epileptic symptoms were present. Including this case as well, no significant change in SIC activity could be found (1.79 ± 0.96 pC/min in control and 368.02 ± 367.8 pC/min with GT949; *p* = 0.17; *n* = 7).

**Figure 4:**
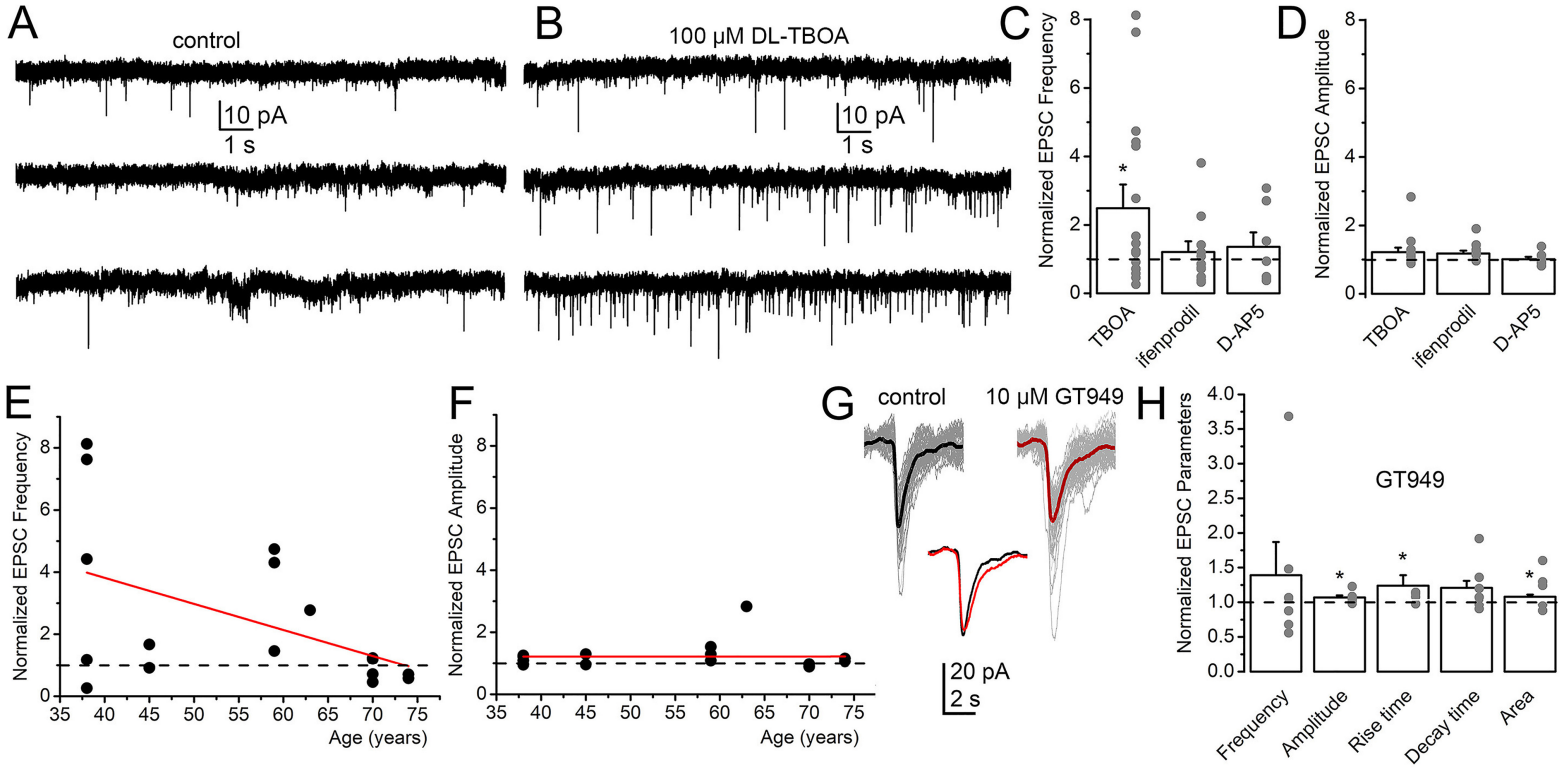
Pharmacological influences on EAATs were capable of altering excitatory postsynaptic current (EPSC) parameters. **(A,B)** Representative current traces under control (magnesium-free) conditions **(A)** and with 100 μM DL-TBOA **(B)**. The negative spikes are the EPSCs. **(C,D)** Statistical analysis of the EPSC frequency **(C)** and amplitude **(D)**. DL-TBOA application led to an almost 2.5-fold increase in frequency, whereas the amplitude was not affected. Columns: average ± SEM, gray dots: individual data points. Dashed line: no change. **(E)** Increase in EPSC frequency by DL-TBOA was age-related. Black dots: individual data, red line: linear fit (*p* = 0.0453, *r*^2^ = 0.24). **(F)** No age-related change was observed in EPSC amplitude (*p* = 0.987, *r*^2^ < 0.0001). **(G)** Changes in EPSC parameters by 10 μM GT949. Black trace: representative average of 50 EPSCs under control conditions, red trace: representative average of 50 EPSCs with GT949, gray traces: superimposed images of 50 individual EPSCs, bottom panel: superimposed image of the control average (black) and the average with GT949 administration. **(H)** Statistical analysis of EPSC parameters with GT949 administration. Note that the amplitude, rise time, and area (charge transfer) had a mild but significant increase. Columns: average ± SEM, gray dots: individual data points. *p < 0.05, **p < 0.01, ***p < 0.001.

The impact of the extrasynaptic glutamate uptake on EPSCs was also assessed. DL-TBOA significantly increased the normalized EPSC frequency whereas ifenprodil and D-AP5 reverted these changes (2.49 ± 0.69-fold increase in TBOA, 1.21 ± 0.31-fold change with ifenprodil and 1.36 ± 0.42-fold increase in D-AP5; *n* = 16; Figures 4A–C). In parallel with it, amplitude (Figure 4D), rise- and decay time were not altered; which findings might indicate presynaptic rather than postsynaptic targets of the actions by DL-TBOA. No correlation was seen between the magnitude of the tonic inward current and changes in EPSC frequency. The only correlation found was between the age of the patient and the change in EPSC frequency: the frequency increase by DL-TBOA declined with age (*p* = 0.0453; *r*^2^ = 0.24; Figure 4E). No such correlation was seen with changes of the amplitude (*p* = 0.987; *r*^2^ < 0.0001; Figure 4F).

GT949 did not alter EPSC frequency. However, the amplitude, rise time and area of the events showed minor but significant increase (1.07 ± 0.03, *p* = 0.031; 1.08 ± 0.03, *p* = 0.013; 1.21 ± 0.1-fold increase, *p* = 0.035, respectively; Figures 4G,H).

## Discussion

In this project we demonstrated that –besides the phasic SICs mediated by GluN2B-subunit containing NMDARs- tonic, NMDAR-mediated inward currents are also elicited by the inhibition of the glutamate uptake. These tonic currents were not (exclusively) mediated by GluN2B subunit containing receptors and absent under control conditions. They also led to an increase in EPSC frequency but no change of the amplitude was revealed. Similar to SICs, tonic inward currents were subject to age-dependent decline.

There is a limited number of studies using human brain samples which detect consequences of neuronal extrasynaptic glutamate receptor activation. The presence of SICs on human neurons was first demonstrated by Navarrete et al. (2013). In our previous study, SICs recorded on human and murine neocortical samples were compared and found that the charge transfer by SICs is significantly greater in humans than in mice. The age-related changes were also strikingly different: in humans, SICs disappeared above a certain age, whereas only a mild reduction in their frequency was seen in mice (Csemer et al., 2023).

Tonic inward currents elicited by extrasynaptic glutamate are present in several CNS areas [as the hippocampus (Fellin et al., 2006; Le Meur et al., 2007; Papouin et al., 2012), the cerebellum (Sasaki et al., 2012), the supraoptic nucleus (Fleming et al., 2011) and the pedunculopontine nucleus (Kovács et al., 2017)]. These inward currents were elicited by inhibition of glutamate uptake (Angulo et al., 2004; Jabaudon et al., 1999; Le Meur et al., 2007; Nie et al., 2010; Fleming et al., 2011; Sasaki et al., 2012). Although all of them were consequences of the increase in extrasynaptic glutamate levels, different glutamate receptors mediated them. AMPAR-dependent tonic inward currents were seen in the cerebellum (Sasaki et al., 2012), whereas mGluR II-mediated currents were found in the pedunculopontine nucleus (Kovács et al., 2017). Our observation is in line with the findings on the supraoptic nucleus and the hippocampus where NMDAR-mediated tonic inward currents were detected (Fellin et al., 2006; Angulo et al., 2004; Jabaudon et al., 1999; Le Meur et al., 2007; Papouin et al., 2012; Fleming et al., 2011). Compared to these previous studies on rodents, the novelty of the current study is that the tonic, NMDAR-mediated inward current elicited by TBOA application was first demonstrated on human tissue.

Although there are important exceptions, it is thought that the GluN2B-containing NMDAR is typically extrasynaptic (Paoletti et al., 2013; Stroebel et al., 2018). We and others demonstrated that SICs elicited by astrocytic glutamate release and NMDAR activation, are largely diminished or fully abolished by GluN2B-specific NMDAR antagonists (Csemer et al., 2023; Zhang et al., 2019; Pirttimaki et al., 2017). However, the subunit identity of the NMDARs mediating the tonic inward currents is not necessarily the same as the ones for SICs. In a study on the hippocampus, GluN2B-containing NMDARs had no contribution to the tonic inward current, which was instead mediated by GluN2C and –D-, and to a lesser extent, GluN2A subunit containing receptors, resembling the synaptic NMDAR profile (Le Meur et al., 2007). In contrast, other recordings on the hippocampus and the supraoptic nucleus revealed a similar subunit profile as our study: the tonic inward current was partially but not fully mediated by GluN2B-containing NMDARs (Fleming et al., 2011; Papouin et al., 2012).

Although SICs and tonic inward currents are both elicited by the ambient glutamate, there might be several differences between the two phenomena. The diffusion of ambient glutamate from its release site to the receptors might distinguish between the phenomena: if the diffusion pathway for glutamate is short enough, SICs are elicited, if the opposite happens, neurons are prone to display tonic currents as a summary of distant SICs (Angulo et al., 2004; Syková and Vargová, 2008). Although glutamate release sites and diffusion pathways were not investigated by us but other differences were also demonstrated between the two phenomena. The receptor identity in the background of them was not identical (see above) and another intriguing difference was also seen. TBOA application led to a baseline ‘noise’ with a low frequency and a moderate amplitude. This phenomenon was reverted by D-AP5 indicating that it might be the consequence of stochastic NMDAR opening rather than technical problems. By eye, the increased oscillatory activity characteristic for the declining phase of SICs was also similar. However, the power spectrum analysis revealed that both the frequency and the amplitude of the latter oscillations are significantly greater, leading to the hypothesis that SICs might have other NMDARs with different activation (or even inactivation-) patterns and these two phenomena might be clearly separable from each other. A combined morphological and functional study would better separate them; revealing the exact locations of receptors for SICs and tonic currents on neuronal processes.

In contrast to SICs, which was seen on neurons under situations close to physiological, we did not find a tonic inward current on pyramidal neurons recorded in naCSF. When the magnesium was omitted from the recording solution, a drastic increase of SIC frequency and charge transfer was seen (Csemer et al., 2023), whereas no inward current was elicited. Similarly, when the uptake of ambient glutamate was facilitated with GT949, the expected appearance of a tonic outward current (as the disappearance of the inward current) did not happen. We therefore concluded that there is no NMDAR-dependent tonic inward current under physiological conditions in the human neocortex. This hypothesis is in line with the literature findings, as administration of TBOA led to seizures (Montiel et al., 2005), as well as the conditional deletion of EAAT2 in astrocytes (Petr et al., 2015). Our findings are in contrast with studies on rodents. There are studies using rodent samples which demonstrated the presence of a tonic NMDAR-mediated current under control conditions in the hippocampus (Le Meur et al., 2007; Papouin et al., 2012) and in the substantia nigra (Wild et al., 2015); which we did not find in the human neocortex.

Tonic neuronal depolarization elicited by increased extrasynaptic glutamate contributes to several neurological diseases as ischemic stroke, traumatic brain injury and the consequential cortical spreading depolarization. The latter phenomenon is a depolarizing wave of cortical networks elicited by increased extracellular K^+^ and glutamate concentrations (Kramer et al., 2016). Glutamate increase is largely due to astrocytic swelling and consequential alterations of glutamate uptake and release, as well as changes in the volume of the extracellular space; although neuronal non-synaptic glutamate release and disturbances of the blood–brain barrier can also be involved (Kimelberg, 2005; Song and Yu, 2014; Dorsett et al., 2017; Frank et al., 2026). In brain malignancies of glial origin, a similar increase in extracellular glutamate is seen (Takano et al., 2001). A typical receptor involved in pathological tonic depolarization is the GluN2B-containing extrasynaptic NMDAR (Hardingham and Bading, 2010; Brassai et al., 2015); although other receptors (e.g., mGluRs in cooperation with NMDARs) might be also involved (Bianchi et al., 2009; O'Connor et al., 1994).

As NMDAR-mediated tonic inward currents were not identified by us under control conditions but with the inhibition of astrocytic glutamate uptake, we hypothesize that this tonic inward current is not a physiological mediator of the human neocortical excitability, rather a contributor to neurological diseases as cortical spreading depolarization or tumor-related hyperexcitability.

Besides tonic inward currents and SICs, changes in EPSCs were evaluated as well. DL-TBOA application led to a greater than 2-fold increase in EPSC frequency, whereas the amplitude did not change. The EPSC frequency increase –similar to the extrasynaptic alterations- was age-dependent. This separate increase in EPSC frequency might implicate solely presynaptic mechanisms (Kerchner and Nicoll, 2008). We hypothesize that this ‘presynaptic’ action might mean a general increase in neuronal excitability and firing rate on the synaptic partners of the recorded neurons, which in turn increase the EPSC frequency recorded on the target neuron (Kerchner and Nicoll, 2008). In contrast to our work on SICs –where the role of these events in synaptic plasticity was demonstrated (Csemer et al., 2023)- the impact of tonic NMDA currents on synaptic plasticity was not investigated in the present study.

Intriguingly, the amplitude, rise time and charge transfer of EPSCs were significantly but modestly increased by application of GT949, whereas the frequency was unaffected. This might imply the existence of postsynaptic modulatory effects by the ambient glutamate inhibited by the artificial lowering of the extrasynaptic glutamate.

We found that there is a decline in both SIC activity and tonic inward current amplitude with aging. These findings can be only partially explained by the detected decline of GluN2B subunit expression (Csemer et al., 2023). Several other factors as decreased expression of EAAT transporters or changes in their functions (Potier et al., 2010; Sharma et al., 2019), as well as astrocytic morphological changes altering the diffusion pathways for glutamate might be in the background (Robillard et al., 2016; Popov et al., 2021; Syková and Vargová, 2008); which needs further investigation.

Interpretation of our results might face with further limitations. First, the human samples are ‘quasi-control’ tissues meaning that most samples are from the vicinity of a tumor which alters conditions of the glutamate homeostasis. Various medications and operative conditions can also affect our results. Age-related limitations are also present as cases younger than 38 and older than 79 are missing which also limits the conclusions drawn. In addition, samples were taken from different cortical areas with known differences (Radnikow and Feldmeyer, 2018; Luebke, 2017).

In conclusion, we hypothesize that tonic NMDAR-mediated inward currents are not parts of physiological regulatory mechanisms on the excitation of the human brain, rather contributors to pathological conditions. Increase of extrasynaptic glutamate leads to excitotoxicity, forming one of the ‘final common pathways’ in the pathogenesis of neurological diseases (Pál, 2018). The inward current demonstrated here can contribute to ischemic current in cortical spreading depolarization. This hypothesis is in line with the previous findings that the vulnerability of the cortex by this phenomenon is decreased with aging (Hertelendy et al., 2019).

## Data Availability

The original contributions presented in the study are included in the article/supplementary material, further inquiries can be directed to the corresponding author/s.
